# The Insertion and Transport of Anandamide in Synthetic Lipid Membranes Are Both Cholesterol-Dependent

**DOI:** 10.1371/journal.pone.0004989

**Published:** 2009-03-30

**Authors:** Eric Di Pasquale, Henri Chahinian, Patrick Sanchez, Jacques Fantini

**Affiliations:** Université Paul Cézanne (Aix-Marseille 3), Université de la Méditerranée (Aix-Marseille 2), Centre de Recherche en Neurobiologie et Neurophysiologie de Marseille, CNRS UMR 6231, INRA USC 2027, Interactions Moléculaires et Systèmes Membranaires, Faculté des Sciences Saint-Jérôme, Marseille, France; University of Portsmouth, United Kingdom

## Abstract

**Background:**

Anandamide is a lipid neurotransmitter which belongs to a class of molecules termed the endocannabinoids involved in multiple physiological functions. Anandamide is readily taken up into cells, but there is considerable controversy as to the nature of this transport process (passive diffusion through the lipid bilayer vs. involvement of putative proteic transporters). This issue is of major importance since anandamide transport through the plasma membrane is crucial for its biological activity and intracellular degradation. The aim of the present study was to evaluate the involvement of cholesterol in membrane uptake and transport of anandamide.

**Methodology/Principal Findings:**

Molecular modeling simulations suggested that anandamide can adopt a shape that is remarkably complementary to cholesterol. Physicochemical studies showed that in the nanomolar concentration range, anandamide strongly interacted with cholesterol monolayers at the air-water interface. The specificity of this interaction was assessed by: i) the lack of activity of structurally related unsaturated fatty acids (oleic acid and arachidonic acid at 50 nM) on cholesterol monolayers, and ii) the weak insertion of anandamide into phosphatidylcholine or sphingomyelin monolayers. In agreement with these data, the presence of cholesterol in reconstituted planar lipid bilayers triggered the stable insertion of anandamide detected as an increase in bilayer capacitance. Kinetics transport studies showed that pure phosphatidylcholine bilayers were weakly permeable to anandamide. The incorporation of cholesterol in phosphatidylcholine bilayers dose-dependently stimulated the translocation of anandamide.

**Conclusions/Significance:**

Our results demonstrate that cholesterol stimulates both the insertion of anandamide into synthetic lipid monolayers and bilayers, and its transport across bilayer membranes. In this respect, we suggest that besides putative anandamide protein-transporters, cholesterol could be an important component of the anandamide transport machinery. Finally, this study provides a mechanistic explanation for the key regulatory activity played by membrane cholesterol in the responsiveness of cells to anandamide.

## Introduction

Anandamide (arachidonoylethanolamide) is a lipid neurotransmitter belonging to the family of endocannabinoids and involved in the regulation of almost all the physiological functions studied including the nervous, the cardiovascular, the respiratory, or the reproductive systems [1]. The intracellular metabolism of anandamide has been the subject of numerous studies. It is well known that anandamide is produced from membrane phosphatidylethanolamine by a two-step reaction catalysed by membrane N-acyl transferase and phospholipase D [2]. Then, intracellular anandamide breakdown by a membrane fatty acid amide hydrolase (FAAH) stops its biological activity [3]. Because of its high lipophilicity together with its amphipathic nature anandamide is generally considered to exert its biological effects in membrane bilayers [4]. In this respect, two scenarios could be proposed. First, anandamide could act directly in the physicochemical environment of the cell membrane which has produced it (autocrine membrane-bound effect). However, anandamide can also be released in the aqueous extracellular space, where, probably transported by a protein carrier, it can reach and stimulate neighbouring cells (paracrine effect). Anandamide attachment to cannabinoid protein receptors, occurs through binding sites deeply embedded in the lipid bilayer [4], [5]. This implies that anandamide has to gain entry into the membrane to stimulate these receptors, which belong to the family of G protein-coupled receptors with seven transmembrane domains.

Interestingly, molecular modeling studies have shown that anandamide could generate either condensed hairpin or extended rod-like structures [6]. This is consistent with a major conformational adjustment of anandamide structure during the reversible transfer of the lipid neurotransmitter from a polar aqueous phase to an apolar lipid phase. Recently, Tian et al. have carefully studied the conformation, location, and dynamic properties of anandamide in a dipalmitoyl phosphatidylcholine (DPPC) multilamellar model membrane bilayer system [4]. They concluded that the neurotransmitter can interact with phospholipids and then laterally diffuse within the bilayer until reaching the transmembrane domains of cannabinoid receptors. However, several important features of anandamide-lipid interactions remained to be explored. In particular, the role of cholesterol in the interaction of anandamide with biological membranes has not been elucidated. Several aspects of anandamide function seem to involve membrane cholesterol. Maccarone and co-workers showed that cholesterol enrichment of glioma cell membranes significantly increased the uptake of anandamide [7]. Reciprocally, depletion of membrane cholesterol in hepatocytes membranes decreased their responsiveness to anandamide [8]. Moreover, lipid rafts, which are enriched in cholesterol, have been involved in the uptake and recycling of anandamide [9]. Altogether, these data strongly suggested that cholesterol could play a pivotal role in the interaction of anandamide with biological membranes and thus in its neurotransmitter activity. There are several ways by which membrane cholesterol could affect anandamide functions. Recently, Bari and co-workers reported that cholesterol modulates the binding efficiency of the endocanninoid receptor CB1, and that this effect is critical for anandamide-induced apoptosis [10]. However, in some cells, anandamide exhibited an apoptotic effect which did not involve the cannabinoid receptors identified so far, but were definitely cholesterol-dependent [8], [11]. Whether these latter effects are mediated by alternative protein receptors functionally coupled to cholesterol, or by cholesterol itself through a protein-independent mechanism, remains to be established. Similarly, no consensus has been reached as to whether anandamide transport through biological membranes requires a protein transporter or not [12].

As stated above, anandamide is a lipid molecule which has to be inserted in the plasma membrane to exert its neurotransmitter activity. As a matter of fact, a simple way for cholesterol to control anandamide functions would be to specifically bind to anandamide, thereby controling its membrane insertion. In particular, a physical interaction between cholesterol and anandamide would explain why cholesterol appears so critical for many aspects of endocannabonoid functions, including anandamide uptake and recycling [9], CB1 activity [10], and receptor-independent apoptosis [8]. However, this possibility has not been experimentally tested. In the present study, we have investigated the behaviour of anandamide in various membrane environments with or without cholesterol, using both monolayer and bilayer membrane models systems. All the lipids used in these experiments were synthetic, so that potential interference with undesirable membrane contaminants could be totally ruled out. We show that anandamide interacts with cholesterol in both monolayer and bilayer systems. We also show that cholesterol triggers the transmembrane transport of anandamide. In complete agreement with these physicochemical results, molecular modeling simulations suggested that cholesterol has a remarkable fit for anandamide, explaining the striking preference of anandamide for cholesterol among other major membrane lipids such as phosphatidylcholine. Thus, whatever the mechanisms by which anandamide triggers a biological effect, cholesterol is able to regulate these effects because it binds to anandamide in the membrane environment.

## Results

### Molecular modeling of anandamide bound to cholesterol

**Figure 1:**
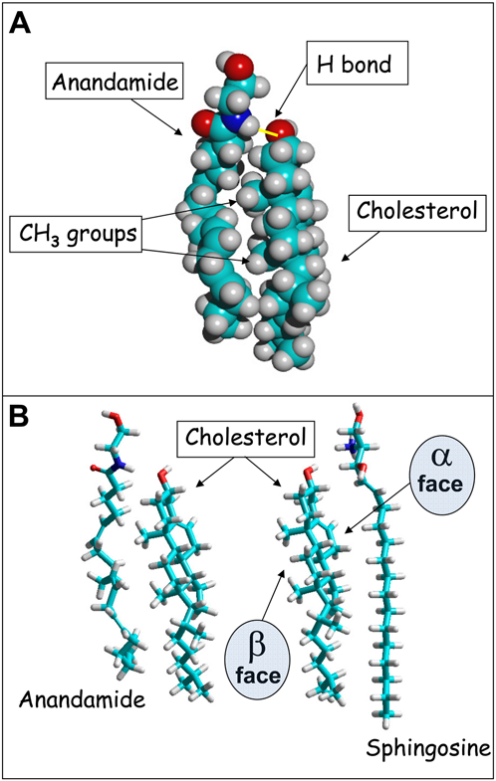
Molecular modeling of anandamide-cholesterol interactions. A- Spacefill model of a cholesterol-anandamide complex in vacuo obtained with the Polak-Ribiere algorithm (for geometry optimization) and then submitted to Monte Carlo simulations. The extended conformation of anandamide in membrane environments is consistent with the study of Tian *et al.* [4]. The methyl groups on the β face of cholesterol fit particularly well with the cavities generated by the double bonds of anandamide. The complex is stabilized by van der Waals interactions and a hydrogen bond (yellow) between the OH group of cholesterol (acceptor group) and the NH group of anandamide (donor group). The total energy of the system is 48.12 kcal.mol^−1^ (−2.81 kcal.mol^−1^ for anandamide bound to cholesterol). B- Comparison of cholesterol-anandamide and cholesterol-sphingosine interactions. The cholesterol-anandamide complex (left panel) is the same as in (A), but shown in a tube representation. The cholesterol-sphingosine complex [13] is shown in the left panel. Note that sphingosine has a good geometric and chemical compatibility with the smooth α face of cholesterol. Anandamide reacts similarly with cholesterol but with its rough β face. The total energy of the system is 48.68 kcal.mol^−1^ for the cholesterol-sphingosine complex (−2.41 kcal.mol^−1^ for sphingosine bound to cholesterol).

In membrane environments, anandamide adopts a typical extended conformation, with its headgroup at the level of the polar headgroup of phospholipids and its terminal methyl group near the bilayer centre [4]. Molecular modeling simulations of anandamide in vacuo led to a quite similar conformation of the lipid (**Figure 1A**). When cholesterol was positioned in the vicinity of anandamide, Monte Carlo simulations indicated that both lipids progressively changed their conformation to find a remarkable complementary fit. The resulting anandamide-cholesterol complex is stabilised by van der Waals interactions between their apolar parts and by a hydrogen bond between the OH group of cholesterol and the NH group of anandamide. This molecular complex respects the orientation of both lipids in biological membranes. Although the extended conformation of anandamide is consistent with an interaction with phospholipids [4], the perfect fit between cholesterol and anandamide suggested a preferential interaction of anandamide with cholesterol rather than with phosphatidylcholine. Since we previously showed that cholesterol also interacts with sphingosine [13], it was interesting to compare the anandamide-cholesterol complex with the cholesterol-sphingosine one. As shown in **Figure 1B**, anandamide interacts with the β face of cholesterol which, according to the nomenclature proposed by Rose and co-workers [14] is the ‘rough’ face with the protruding methyl groups. In contrast, sphingosine binds to the ‘smooth’ α face of cholesterol. The respective energies calculated for the cholesterol-anandamide and cholesterol-sphingosine complexes are remarkably similar (see **Figure 1** legend for details). As biochemical studies have confirmed that sphingosine actually interacts with cholesterol [13], these data suggest that the interaction between anandamide and cholesterol is also very likely.

**Figure 2:**
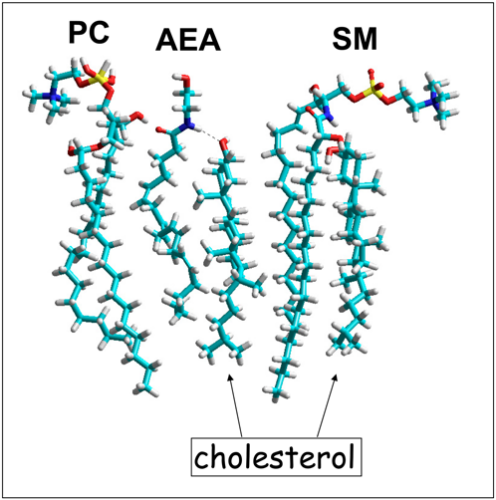
Molecular modeling of the anandamide-cholesterol complex in the membrane environment. The anandamide (AEA)-cholesterol complex was generated in vacuo, and subsequently merged with a phosphatidylcholine molecule (PC). The flexible acyl chains of PC could satisfactorily fit with AEA, allowing PC to interact with the AEA-complex. A molecule of sphingomyelin (SM) interacting with cholesterol was added to the system as a representative lipid raft component. The SM-cholesterol complex could also fit with the AEA-cholesterol complex. Namely, in the AEA-cholesterol complex, the α face of cholesterol is fully accessible for the sphingosine chain of SM. This molecular modeling study indicates that AEA can bind to cholesterol in both the PC-rich fluid (Ld) phase and the SM-rich (Lo) phase of the plasma membrane. In particular, the H-bond between cholesterol and AEA is still present after introducing these membrane lipids in the system. The total energy of the system is 180.68 kcal.mol^−1^ for the cholesterol-sphingosine complex (−14.43 kcal.mol^−1^ for AEA bound to cholesterol). All models were obtained with the Polak-Ribiere algorithm (for geometry optimization) and then submitted to Monte Carlo simulations.

However, anandamide is a very flexible molecule whose conformation might be strongly influenced by its environment [4]. For these reasons, we studied the impact of vicinal membrane lipids on the strength of the anandamide-cholesterol interaction. Phosphatidylcholine, a representative lipid of the liquid disordered (Ld) phase of the plasma membrane [15] was injected first in the system. As shown in Figure 2, this lipid accommodated its conformation to the shape of anandamide, and it had virtually no impact on the anandamide-cholesterol complex. Then a sphingomomyelin molecule in tight interaction with cholesterol [15] was introduced in the system as a representative component for the liquid ordered (Lo) phase, which corresponds to lipid raft domains [16]. Again, these lipids did not destabilize the anandamide-cholesterol complex (**Figure 2**). Sphingomyelin readily bound to the α face of cholesterol which is still fully accessible when the sterol interacts with anandamide. Overall these data suggested that a specific interaction between anandamide and cholesterol could take place in both the Ld and the Lo phases of the plasma membrane. Physicochemical experiments were then conducted to assess whether anandamide could actually interact with cholesterol in membrane environments.

### Interaction of anandamide and structurally related compounds with lipid monolayers

In a first series of experiments, we prepared various lipid monolayers at the air-water interface and measured the interaction of anandamide with these synthetic half-membranes using the Langmuir film balance technology [13], [17]. Anandamide (50 nM) was injected in the aqueous subphase underneath the monolayer and its interaction with the lipids was detected by a surface pressure increase. This increase in the surface pressure is caused by the insertion of anandamide molecules between the lipids of the monolayer. It is a direct and quantitative measurement of the interaction of a ligand (anandamide in this case) with a given lipid [13]. Therefore, the higher the value of the surface pressure increase induced by anandamide, the higher the affinity of anandamide for a given lipid monolayer.

**Figure 3:**
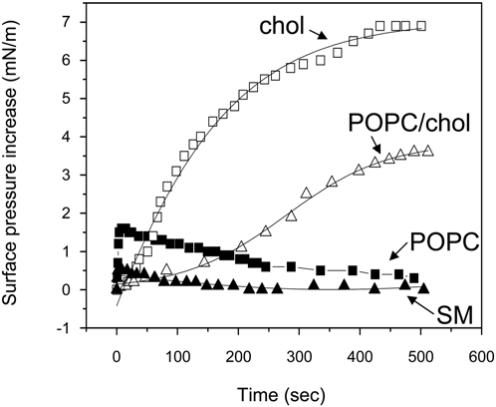
Interaction of anandamide with various lipid monolayers. The indicated lipid was spread at the air-water interface to form a compressible monolayer at an initial pressure comprised 20–25 mN.m^−1^. After stabilization of the monolayer, anandamide was added in the aqueous subphase (final concentration 50 nM). Under these specific conditions, anandamide does not modify the surface tension of water (not shown), so that it will have no effect on the surface pressure by itself. Thus, any change in the surface pressure indicates an interaction of anandamide with the lipid monolayer. The data are expressed as the variation of the surface pressure as a function of time following the addition of anandamide. The following lipids were spread at the air-water interface: pure POPC (full squares), pure cholesterol (open squares), mixed POPC-cholesterol film (1∶2, mol∶mol, open triangles), pure SM (full triangles).

With this in mind, one can see in **Figure 3** that anandamide interacted more efficiently with cholesterol than with palmitoyl-oleyl-phosphatidylcholine (POPC), a physiologically relevant glycerophospholipid. Interestingly, the interaction with cholesterol progressively increased over time until reaching a plateau value (+7 mN.m^−1^) after 7 minutes. This value is remarkably close to the one of +6 mN.m^−1^ obtained upon the addition of sphingosine (50 nM) under a monolayer of cholesterol [13]. In contrast, following a very rapid initial increase of surface pressure induced by anandamide, the pressure of the POPC monolayer progressively decreased to eventually return to the initial value. This effect, which was reproducibly observed over six independent experiments, suggests a decrease in the area of the monolayer, which could be due to the extraction of either anandamide, or of anandamide-POPC complexes, or of POPC alone. Moreover, anandamide interacted with mixed cholesterol-POPC films, but not with sphingomyelin (SM). Clearly, our data revealed a striking selectivity of anandamide among major membrane lipid species with a definitive preference for cholesterol.

**Figure 4:**
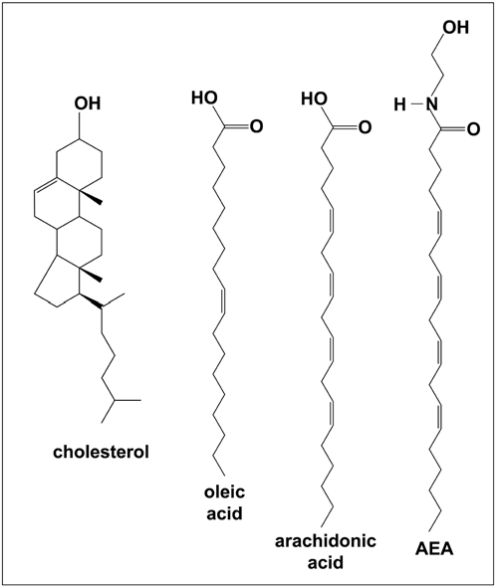
Chemical structures of cholesterol, anandamide and unsaturated fatty acids. Unsatutared fatty acids (arachidonic acid and oleic acid) are structurally related to anandamide. The interaction of cholesterol with these fatty acids, in comparison with anandamide, is shown in Figure 5.

**Figure 5:**
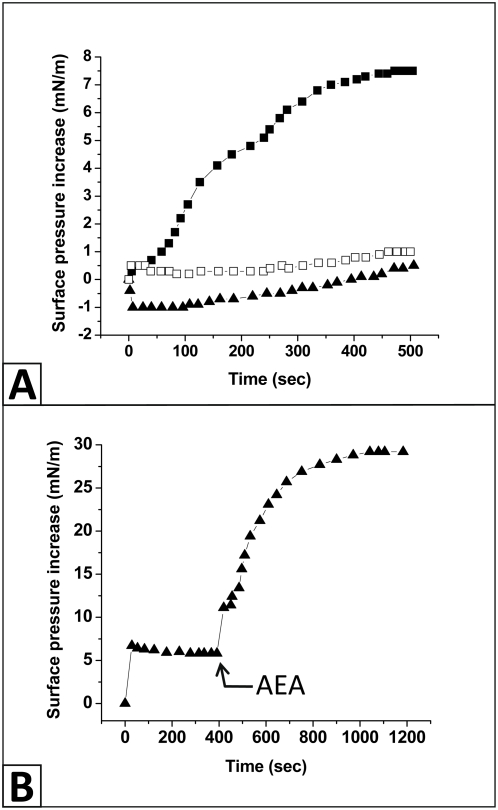
Interaction of arachidonic acid, oleic acid and anandamide with cholesterol monolayers. *A*. Monolayers of pure cholesterol were prepared at the air-water interface at an initial pressure of 25 mN.m^−1^. After stabilization of the monolayer, arachidonic acid (open squares), oleic acid (full triangles) or anandamide (full squares) were injected in the aqueous subphase at a final concentration of 50 nM. The data are expressed as the variation of the surface pressure as a function of time following the addition of the indicated lipid in the subphase. *B*. A monolayer of cholesterol (initial pressure of 25 mN.m^−1^) was incubated with 750 nM of oleic acid added in the subphase. Under these conditions, the surface pressure of the monolayer rapidly increased and reached a stable value of 5 mN m^−1^. After 400 seconds, 750 nM of anandamide (AEA, arrow) was injected in the aqueous subphase, which induced a dramatic surface pressure increase.

An important issue was to assess whether similar results could be obtained if structurally related lipids were assayed instead of anandamide. Anandamide is metabolically derived from a phospholipid precursor containing an unsaturated acyl chain derived from arachidonic acid [2]. Thus we studied the interaction of two unsaturated fatty acids, i.e. arachidonic acid and oleic acid, with cholesterol monolayers. The molecular structures of these compounds are shown **in** **Figure 4**. Both oleic and arachidonic acids could fit with the apolar part of cholesterol and establisha H-bond with its OH group. However, arachidonic acid (50 nM) induced only a very weak increase in the surface pressure (+1 mN.m^−1^ after 500 seconds, compared with +7.5 mN.m^−1^ for anandamide) (Figure 5A). Oleic acid (50 nM) induced an immediate decrease in the surface pressure (−1 mN.m^−1^ after 5 seconds) followed by a very weak and progressive increase (+0.5 mN.m^−1^ after 500 seconds). These data showed that in the nanomolar concentration range, anandamide but neither oleic acid nor arachidonic acid did interact with cholesterol monolayers. To observe an interaction equivalent to the one induced by 50 nM anandamide, the concentrations of arachidonic acid and oleic acid had to be raised respectively to 500 nM and 750 nM. The interaction of oleic acid (750 nM) with a cholesterol monolayer is shown in Figure 5B. The surface pressure first increased (+7 mN.m^−1^) shortly after the addition of oleic acid, after which the surface pressure progressively decreased to reach a stable value of 5 mN.m^−1^ after 400 seconds of incubation. At this time, we injected an equivalent concentration of anandamide (750 nM) to compare the effects of both lipids on the same cholesterol monolayer. The surface pressure was dramatically increased upon addition of this concentration of anandamide in the subphase until reaching a stable plateau value (+23.5 mN.m^−1^) after 800 seconds. Overall, these data showed that unsaturated fatty acids that are structurally related to anandamide do not behave like anandamide when probed on cholesterol monolayers. These results clearly demonstrate that the interaction of anandamide with a pure cholesterol monolayer is specific. Closely related amphipathic molecules such as arachidonic acid and oleic acid can also interact with cholesterol, but at significantly higher concentrations than anandamide does (10 times and 15 times, respectively). In any case, at all the concentrations tested in this study, the effects of anandamide on cholesterol monolayers were always stronger than those of fatty acids.

**Figure 6:**
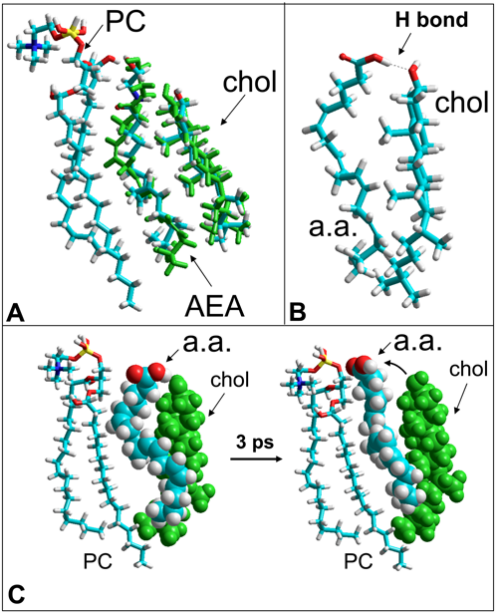
Molecular dynamics simulations of cholesterol-anandamide and cholesterol-arachidonic acid minimized complexes in a membrane lipid environment. A- Geometry optimization of anandamide (AEA) interacting with both cholesterol (chol) and phosphatidylcholine (PC). The molecules with the carbon atoms in blue correspond to the initial conditions (energy minimum complex simulated in vacuo with the Polak-Ribiere algorithm). Molecular dynamics simulations of the AEA-cholesterol complex were then conducted in vacuo with the MM+ force field. The respective positions of AEA and cholesterol after 3 ps of simulation are indicated in green. One can see that AEA is still tightly bound to cholesterol, indicating that PC had little influence on the geometry of the AEA-cholesterol complex. B- Tube representation of a minimized complex between arachidonic acid (a.a) and cholesterol. The model was obtained with the Polak-Ribiere algorithm using the anandamide-cholesterol complex of Figure 1 as template. The H bond between the –COOH group of arachidonic acid and the OH group of cholesterol is indicated as a dot line. C- Spacefill models showing the evolution of the cholesterol-arachidonic acid complex (in presence of a PC molecule shown in a tube representation) after 3 ps of molecular dynamics simulations. The initial conditions are shown in the left panel, and the geometry obtained after 3 ps of simulation in the right panel. The system has spontaneously evolved toward the dissociation of arachidonic acid from cholesterol (arrow). The conformation of arachidonic acid after 3 ps is more fitted to the geometry of the vicinal PC molecule than to the sterol. This suggests that in the membrane environment, the affinity of cholesterol for arachidonic acid is weaker than for anandamide. Consequently, the complex between cholesterol and arachidonic acid is predicted to be less stable than the complex between cholesterol and anandamide.

If one considers the molecular structure of anandamide and arachidonic acid, this can be viewed as paradoxical. Indeed, the polar part of arachidonic acid consists of a carboxylic group, which displays an OH in place of the NH of anandamide. In the anandamide-cholesterol complex shown in **Figure 1**, the hydrogen atom of NH is involved in an H bond with the oxygen atom of cholesterol. As shown in **Figure 6B**, a minimized complex between the protonated form of arachidonic acid and cholesterol can be obtained. This complex was then positioned in front of a phosphatidylcholine molecule to mimic the membrane environment. The short-term evolution of this cholesterol-arachidonic acid complex, constrained by phosphatidylcholine, was studied in silico by molecular dynamics simulations. After 3 ps, arachidonic acid had detached from cholesterol to adopt a conformation more fitted to the apolar flexible chains of phosphatodylcholine (**Figures 6C**). At this time, the hydrogen bond and several van der Waals interactions that were initially involved in the cholesterol-arachidonic acid complex had been broken. For comparison, we studied the evolution of a cholesterol-anandamide complex under similar conditions. In marked contrast with the data obtained with arachidonic acid, the anandamide-cholesterol complex had remained remarkably stable after 3 ps of simulation, with its stabilizing hydrogen bond still operative (**Figure 6A**). These modeling studies, which are in line with the physicochemical data, suggest that cholesterol has a higher affinity for anandamide than for arachidonic acid. This specific issue will be further discussed below.

### Interaction of anandamide with lipid bilayers

To characterise the biophysical mechanisms responsible for cholesterol-dependent anandamide interactions, we prepared planar lipid bilayer membranes [18] of controled biochemical compositions. In this model, a bilayer is formed by injecting with a Hamilton syringe, 1–2 µl of a concentrated lipid solution (POPC or POPC-Cholesterol, 50 mg/ml) in a hole drilled in the wall of a Delrin chamber (200 µm diameter) thus separating two compartments. The formation of a gigaohm-seal characteristic of an operating membrane has been systematically monitored electro physiologically. Ag/AgCl electrodes are used to record the electrical variations induced by the ionic fluxes crossing through the membrane. One electrode is connected to the amplifier and dipped into the Delrin chamber thus defining the cis compartment. The other is connected to the ground and defined the trans compartment. Thus, this experimental setup is adequate to evaluate the insertion of organic compounds within the lipid bilayer.

**Figure 7:**
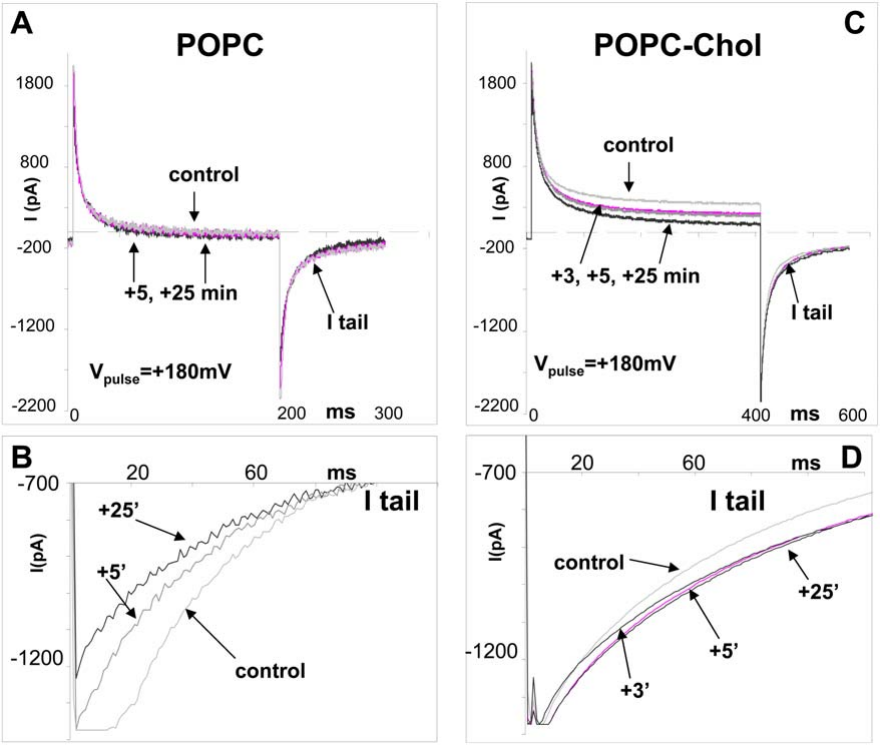
Action of anandamide (AEA, 400 µM) on planar lipid bilayers: effect of cholesterol. *A–B*. superimposed currents across a bilayer constituted of POPC alone (*A*), held at 0 mV, in response to a rectangular pulse (+180 mV, 200 ms), before (control, light grey bilayer alone), and after addition of anandamide (heavy grey, +5, +25 min after AEA injection). *B*. Expanded view of tail currents (I tail) showing their decrease over time. *C–D*. POPC-cholesterol (2∶1, mol∶mol). AEA injection (*C*) stably increased both resistance and capacitance. Maximum effect was obtained for all POPC-cholesterol bilayers within 3–5 min. Each current trace represents the average of 6 records.

**Figure 8:**
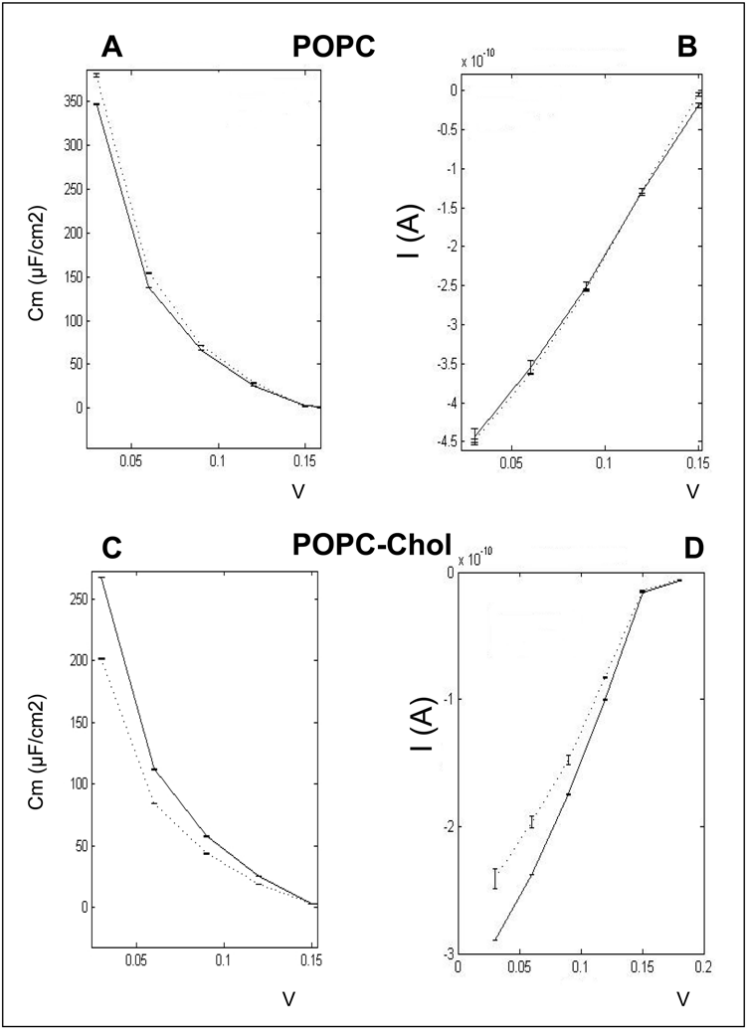
Modification of bilayers electrical properties. *A–D*. dotted line, control; solid line, after AEA injection. Intensity-Voltage curves were obtained from the relaxation tail currents plotted against voltage, and current reading at 225 ms and 425 ms (POPC and POPC-cholesterol respectively). Capacitance-Voltage curves, I_0_ reading was also at 225 and 425 ms, and I_t_ 100 ms later. Error bars were calculated after current acquisitions from six repeated protocols, from the same membrane, and then fed to the analysis programme.

Now, the way that anandamide is transferred from stock organic solution to aqueous buffers is debated among investigators. As a matter of fact, anandamide transport studies, which require higher anandamide concentrations than those we used in the monolayer assay, are generally conducted in presence of bovine serum albumin [19], which improves anandamide solution stability and minimizes its binding to plastic surfaces [12]. However, it has also been reported that serum albumin could generate some artifacts in transport studies [20]. Furthermore, in our hands, bovine serum albumin appeared to be deleterious for planar bilayer membranes (not shown). Finally, the spectroscopic method used for anandamide detection does not allow the use of a protein carrier such as serum albumin. Thus we decided to use methyl-beta-cyclodextrin (MβCD) to prepare aqueous anandamide solutions useable for planar bilayer studies. Indeed, cyclodextrins are well known for their ability to increase substantially the aqueous solubility, stability and bioavailability of lipophilic drugs, so that they are suitable carriers for anandamide [21]. In this respect, it should be noted that only the free drug, and not the cyclodextrin-drug complex can penetrate across the biological membranes [22]. In any case, we carefully controled that under our experimental conditions, 4.2 mM of MβCD alone has no effect on the capacitance and resistance of lipid bilayers (not shown). Then we added anandamide in presence of MβCD in the cis compartment in which a functional membrane of pure POPC had been formed by the injection method [18]. **Figures 7A and 7B** show the resulting currents after a 180 mV voltage pulse. In response to anandamide, there was a transient increase in the bilayer capacitance during the first minute (not shown), followed after 5 minutes by a stable capacitance decrease of 15±8% (*p*<0.05; range: 14–2 µF/cm^2^, n = 7, **Figure 8A**). Therefore, the evolution of this electrical parameter in response to anandamide is fully consistent with the interaction of anandamide with a POPC monolayer (initial increase of the surface pressure followed by progressive decrease as shown in **Figure 3**). Changes in capacitance are due to the variations of the actual membrane surface [18]. The time-course of the electrical resistance of the membrane bilayer evolved similarly, with a decrease of 26±12% (*p*<0.05, range: 4.2×10^4^–0.2×10^4^ ohm-cm^2^, n = 7, **Figure 8B**). From these studies conducted with two distinct model membranes it can be concluded that: i) anandamide interacts with POPC, ii) this interaction is rapidly reversible, and iii) the presence of anandamide in pure POPC phases could modify the phospholipid organisation.

The incorporation of cholesterol in POPC bilayers (**Figures 7C and 7D**) highly stimulated anandamide insertion. Namely, when added on a bilayer with a molar POPC-cholesterol ratio of 2∶1 (mol∶mol), anandamide induced an immediate and permanent increase in capacitance, 19±9% (*p*<0.01, range: 100–8 µF/cm^2^, n = 7, **Figure 8C**), indicating a stable insertion process. This is in line with the results obtained with cholesterol monolayers (**Figure 3**). Interestingly, anandamide also increased the resistance of the membrane by 16±5% (*p*<0.01, range: 3.8×10^4^–4.1×10^4^ ohm-cm^2^, n = 7, **Figure 8D**), which indicated that the integrity of bilayers was not compromised by anandamide.

### Translocation of anandamide through lipid bilayers

**Figure 9:**
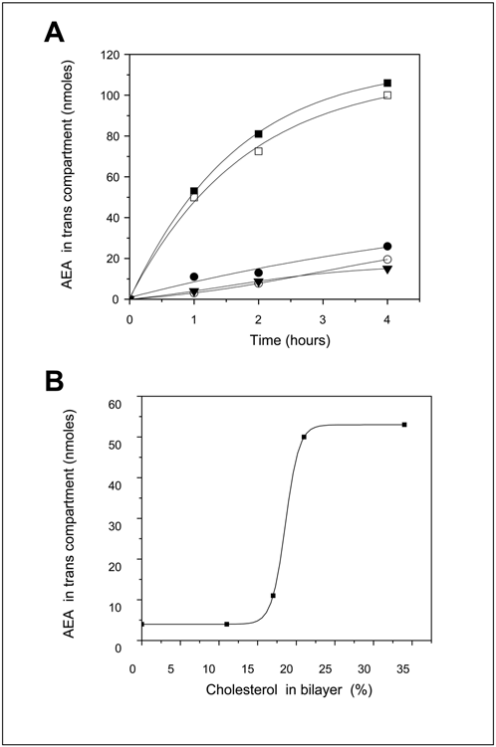
Kinetics of anandamide transport through planar lipid bilayers: effect of cholesterol. Planar lipid bilayers consisting of pure POPC (full triangles), or various mixtures of POPC and cholesterol [molar∶ molar 2∶1 (full squares), 4∶1 (open squares), 5∶1 (full circles), 8∶1 (open circles)] were obtained as described for electrical measurements. *A*. Anandamide (1 mM) was added to the cis compartment in presence of 4.2 mM MβCD. The amount of anandamide recovered in the trans compartment was enzymatically measured using an anandamide assay with 5-lipoxygenase. *B*. Anandamide transport as a function of cholesterol percentage in the POPC bilayer. The experimental points correspond to the amount of AEA in the trans compartment 1 hour after its addition in the cis compartment. The sigmoidal shape of the cholesterol effect suggests that a threshold level of cholesterol is required to trigger the translocation of AEA. This is consistent with the recruitment of cholesterol molecules into tail-to-tail dimers as the ordered supramolecular cholesterol structure used by AEA to cross the membrane. On the opposite, a further excess of cholesterol (full squares, panel A) would condense into non functional nanodomains unable to further increase the kinetics of AEA transport.

The next issue addressed herein was to determine whether cholesterol has any impact on the membrane translocation of anandamide. Using the same bilayer design, we studied the kinetics of anandamide transport through POPC bilayers prepared with various cholesterol amounts. Anandamide (1 mM) was added in the cis compartment of the bilayer chamber in presence of 4.2 mM MβCD. The concentration of anandamide in each compartment at various times was determined by an enzymatic assay based on the 5-lipoxygenase catalysed reaction on cis-cis 1–4 pentadiene structure systems [23] (threshold detection level 2.5 nmol of anandamide). The data in **Figure 9A** clearly shows that pure POPC membranes have a very weak permeability to anandamide. Above a critical threshold level, the presence of cholesterol in the POPC matrix strongly stimulated the translocation of anandamide. For instance, after one hour, cholesterol increased by twelve times the amount of AEA transfer (**Figure 9B**). The electrical parameters of bilayers were assessed by electrophysiological monitoring, so that we can be certain that - under our experimental conditions – the presence of MβCD did not affect the integrity of the bilayers, whatever their cholesterol content (in the range of 8∶1 to 2∶1 POPC∶cholesterol molar ratio). In particular, the increase in membrane capacitance measured upon incubation of MβCD-anandamide complexes with cholesterol containing lipid bilayers demonstrates that, under these conditions, MβCD does not extract cholesterol. This is fully consistent with the data independently obtained by Ohvo and Slotte [24] who showed that cyclodextrin-dependent cholesterol extrusion from model membranes requires a high excess of cholesterol vs. phospholipids, which is not the ratio we used in our experiments. Finally, POPC-cholesterol bilayers incubated only with 4.2 mM of MβCD, were totally impermeable to glucose (data not shown), which demonstrated that these membranes were not leaky to small polar molecules.

## Discussion

The main outcomes of the present study can be summarized as follows: i) anandamide can adopt a specific conformation compatible with a high affinity interaction with cholesterol; ii) although anandamide can interact with phosphatidylcholine [25], cholesterol greatly facilitates its insertion into reconstituted model membranes; and iii) the presence of a threshold level of cholesterol is sufficient to induce the translocation of anandamide through a protein-free membrane. Such threshold levels could correspond to local enrichments of the plasma membrane in pure cholesterol which could exist for instance at the boundaries of lipid raft domains [16], [26].

That anandamide could interact with cholesterol has been previously suggested by Biswas *et al.* on the basis of the binding of cholesterol to anandamide-coated polymixin beads [8]. However, this experimental system does not adequately reflect the orientation and organization of cholesterol in a biological membrane. In addition, the lipid specificity of the interaction has not been investigated by these authors, so that we cannot conclude from their study whether anandamide has a general affinity for lipid molecules or a specific fit for cholesterol. In our study, we show that one peculiar anandamide conformation displays a remarkable complementary fit for cholesterol (**Figure 1**). Molecular modeling approaches indicated that anandamide interacts with the β face of cholesterol. Interestingly, previous studies suggested that sphingosine, which also binds to cholesterol, recognizes its α face [13]. These geometric features allow cholesterol to interact simultaneously with anandamide and with sphingolipids. Moreover, the energy calculations of cholesterol-anandamide and cholesterol-sphingosine interactions appeared to be remarkably similar, suggesting highly specific interactions in both cases. The surface pressure values measured during the interaction of cholesterol monolayers with anandamide and with sphingosine were also fully consistent (ca. +6–7 mN.m^−1^ for the concentration of 50 nM for both sphingosine and anandamide).

These data, obtained with lipid monolayers, were fully confirmed by the physicochemical studies of bilayer model membranes, which demonstrated that the presence of cholesterol dramatically increased the insertion of anandamide into the lipid phase. It should be noted that the capacitance values obtained with this system were significantly higher than those obtained in comparable studies of synthetic phospholipid bilayers probed with penetrating compounds [27]. This indicates that the cholesterol stimulation of anandamide insertion into bilayer membranes is a not a borderline phenomenon but a quantitatively significant process.

As the bilayer system is particularly well suited for transport studies, we could also analyze the effect of cholesterol on the translocation of anandamide. Our data clearly showed that bilayers consisting of POPC alone were only weakly permeable to anandamide. This agreed with the low affinity of anandamide for POPC measured in both monolayer and bilayer systems. However, in presence of a threshold level of cholesterol, the transport of anandamide through the synthetic membrane was greatly enhanced. On this basis, one could hypothesize that anandamide could interact chiefly with cholesterol enriched domains such as lipid rafts [9], and especially the boundaries of these domains where local cholesterol clusters could modulate the interaction of these domains with the bulk membrane [16], [26].

Until now, the mechanism of anandamide transport through a biological membrane is matter of debate [12], 28. According to some authors, anandamide passively diffuses through the membrane, its transmembrane gradient being maintained by the intracellular action of FAAH which hydrolyses anandamide into arachidonic acid and ethanolamine [29]. Because the transport of anandamide is saturable and specifically inhibited by anandamide analogues that are not broken by FAAH, other authors argue for the existence of a proteic transporter for anandamide [30]. An important specificity of the present study is that the different model membranes used (monolayers and bilayers) were built with synthetic lipids. Accordingly, we could totally rule out the possibility that some of the observed effects could be due to protein contaminants. Indeed, our data clearly indicate that cholesterol has the intrinsic property to stimulate both the insertion and transmembrane transport of anandamide. Thus, even though a putative protein anandamide transporter can exist, clearly this latter is not compulsory. Further studies will help to clarify the role of membrane cholesterol in anandamide transport, especially its saturability and sensitivity to anandamide analogues.

Our data are also consistent with the observation that the uptake of anandamide is inhibited by cholesterol depleting agents, and stimulated by membrane cholesterol enrichment [7]. The ability of anandamide to recognize and bind to membrane cholesterol could also explain why anandamide uptake and/or biological effects are modulated by pharmacological agents that affect the homeostasis of lipid rafts (cholesterol-enriched microdomains) [9], [10]. As for the effects of anandamide that are mediated by its well-characterized protein receptors such as CB1 [8], cholesterol could exert two distinct (yet not mutually exclusive) functions: i) modulation of receptor conformation with an impact on ligand binding efficiency, and ii) facilitation of the transfer of anandamide to the protein receptor following its insertion into the membrane. Finally, the cholesterol-anandamide interaction could also be responsible for the effects of anandamide that do not appear to involve its classical transmembrane protein receptors [8], [11], [31].

An intriguing and perhaps unexpected aspect of the interaction of anandamide with cholesterol is its relative specificity. We have shown that unsaturated fatty acids structurally related to anandamide do not interact with cholesterol monolayers unless their concentration is raised to 500 nM for arachidonic acid (10 times higher than anandamide) and 750 nM for oleic acid (15 times higher than anandamide). This can be interpreted as a better affinity of cholesterol for anandamide than for unsaturated fatty acids such as the arachidonic acid. The apolar parts of arachidonic acid and anandamide are identical. The difference lies in the polar part, which is a carboxylic group for the fatty acid and ethanolamide for anandamide. As a matter of fact, the ethanolamide group has a marked impact on the stability of the anandamide-cholesterol complex, as suggested by molecular dynamics simulations. Yet the low activity of arachidonic acid on cholesterol monolayers could also be due to the dissociation equilibrium of the carboxylic group. The pKa of this group for polyunsaturated fatty acids is close to pH 8, but is known to fall towards pH 7 for diluted solutions [32]. Thus, under the experimental conditions of the monolayer experiments, arachidonic acid might indeed be a balanced mixture of negatively charged and neutral protonated molecules. Since only the protonated form of the acid could establish an H bond with the oxygen atom of cholesterol, this feature could also explain the instability of arachidonic acid-cholesterol complexes (this effect was not studied in our molecular dynamics simulation). In contrast, anandamide is a neutral molecule and the hydrogen atom of its NH group is not labile. This makes the H bond between anandamide and cholesterol particularly stable.

Overall, our physicochemical data indicated that i) anandamide preferentially interacts with cholesterol in monolayer and bilayer systems (compared with other membrane lipids such as POPC or sphingomyelin), and ii) this effect is specific for anandamide as assessed by the study of structurally related compounds (arachidonic acid and oleic acid).

### Conclusion

Our demonstration of a specific interaction between anandamide and cholesterol has important consequences for the neurobiology of anandamide. The efficient transfer of anandamide from the aqueous phase (i.e. bound to a carrier protein) to the plasma membrane is associated with a reduction of dimensionality from a three-dimensional space to a two-dimensional surface diffusion [33]. Consequently, the concentration of anandamide in the membrane could be of several levels of magnitude higher than in the intercellular space. Since cholesterol is present in both leaflets of the plasma membrane [34], it could trigger the bidirectional translocation of anandamide (according to the concentration gradient of the neurotransmitter). Indeed, anandamide could be transferred from one membrane leaflet to the other by cholesterol dimers adopting a tail-to-tail configuration [34]. This transmembrane transport is still consistent with a passive diffusion mechanism, but involving a cholesterol concentration threshold as illustrated in **Figure 9**. Finally, whether anandamide bound to cholesterol could be laterally transported in the plane of the membrane and delivered to its transmembrane receptors CB1 and CB2 warrants further investigation.

## Materials and Methods

### Materials

Synthetic lipids of the higher purity available were purchased from Sigma. Pure water was from BIORAD. All other reagents were from Sigma.

### Molecular modeling

Geometry optimization of each cholesterol-lipid complex was first achieved with the Polak-Ribiere algorithm. Monte Carlo (**Figures 1** **,** **2**) and molecular dynamics simulations with the MM+ force field (**Figure 6**) were then performed in vacuo with the Hyperchem 7.5 program (ChemCAD, Obernay, France) as described previously [13], [17].

### Surface pressure measurements of lipid monolayers

The surface tension was measured with a fully automated microtensiometer (μTROUGH SX, Kibron Inc. Helsinki, Finland). All experiments were carried out in a controled atmosphere at 20°C±1°C. Anandamide (stock solution prepared in hexane∶chloroform∶ethanol; 11∶5∶4; vol∶vol∶vol and saturated under a nitrogen flux) was injected in the pure aqueous subphase (volume of 800 µl) with a 10 µl Hamilton syringe, and the variations of the surface pressure were continuously recorded until reaching equilibrium. The data were analyzed with the Filmware 2.5 program (Kibron Inc. Helsinki, Finland) as described previously [13], [17]. The accuracy of the system under our experimental conditions was ±0.25 mN.m−1 for surface tension. To compare the effects of anandamide, arachidonic acid and oleic acid, 100× solutions of these lipids were prepared in 100 mM NaCl and injected in the aqueous subphase.

### Reconstituted planar lipid bilayers

Planar lipid bilayers were formed over a 200 µm diameter circular hole, in a vertical wall of a Delrin chamber (Warner instruments), by the injection method [18]. The membrane forming solution consisted of palmitoyl-oleoyl-phosphatidylcholine (POPC) or POPC/cholesterol (2∶1; 4∶1; 8∶1, mol/mol; synthetic lipids purchased from Sigm). The bilayers were obtained by injecting 1 µl of lipids (50 mg/ml in: hexane/chloroform/ethanol, 11/5/4, v/v/v) with a Hamilton syringe. The aperture was not pre-treated before the injection of lipids.

### Electrical recordings

All experiments were performed in 0.1 M KCl, 0.001 M 4-(2-hydroxyethyl)-1-piperazineethanesulfonic acid (HEPES) (pH 7.2; Sigma; H_2_O from Biorad). Each compartment contained 1.4 ml of this solution. An Ag/AgCl electrode connected to a patch-clamp amplifier (Axopatch 200B, Digidata 1200, Axon Instruments) via a probe was immersed (using a glass micropipette, 1–2 GΩ) in the cis compartment, the trans compartment was connected to the ground. The formation of bilayers was electrically monitored by measuring online the membrane apparent resistance (−5 mV/0.5 s). After lipid injection, a gigaohm (GΩ) seal was obtained within seconds. Membranes were maintained at zero mV in voltage clamp mode and typically stabilized at −80 pA in these ionic conditions. Capacity increased with time until a steady-state value some 2–10 min later and considered as membrane stabilization. This was controled by the total reduction of the leak current after a 100 mV- 1 s pulse, probably reflecting the solvent elimination. Electrical apparent resistance ranged 10–40 GΩ for POPC and POPC-cholesterol bilayers. The breakdown potential was estimated around 220–250 mV using rectangular voltage steps. Since fluctuations of currents were typically observed for steps by 200 mV (assessed over a hundred bilayers), the maximum voltage step was set at 180 mV. Experiments were performed at room temperature. One µmole of AEA from an ethanolic stock solution (10 mM) was dried in saturated nitrogen atmosphere then resuspended in 50 µl of methyl-beta-cyclo-dextrin (MβCD) solution (87 mM) and injected in the cis compartment. The same volume of MβCD 87 mM was injected in the trans compartment. Only one injection of AEA was performed per bilayer. Different POPC/cholesterol ratios were tested in the present work (from 16∶1 to 2∶1) and only the 2∶1, corresponding to the most efficient ratio for transport of AEA through bilayers, is described and illustrated in Figure 5. There was no capacitance modification for 16∶1 and 8∶1 ratios. The 4∶1 ratio gave only one minor capacitance variation over ten bilayers. Thus, no graded response was observed from 16∶1 to 2∶1 POPC/cholesterol ratios, suggesting a threshold effect of cholesterol concentration.

#### Data analysis

Off-line analysis was carried out using the subroutine Clampfit of the Pclamp6 software to export data. Membrane resistance and capacitance were calculated using a home-made (with Matlab) computer analysis programme (see below). Electrical resistance was obtained by impressing a family of voltage step across the membrane (30 mV/2 s) and calculated according to the ohm's law R_M_ = (E_M_/E_i_−E_M_)*R_i_ where Ri is the series resistance (stated at 1 MΩ, which is the resistance of a glass pipette), E_i_ is the calibrated input voltage, and E_M_ is the voltage across the bilayer. Current/voltage (I/V) curves were generated from the BLM current responses, as a function of applied voltage. The experimental transmembrane resistance R_M_ (in ohms) was converted to the normalized transmembrane resistance R_m_ (in ohm-cm^2^) by calculating the product of R_M_ times the membrane area (A). Since A is usually expressed in cm^2^, the equation becomes R_m_ = R_M_*A.

R_m_ varied linearly in the 30–60 mV voltage range, in both types of bilayers. From 60 to 180 mV, POPC I/V curves remained linear after a change in slope, whereas POPC-cholesterol bilayers exhibited a slight rectification, and a change in slope. The steady-state current for a 180 mV pulse taken as reference, was obtained around 200 ms and 400 ms for POPC and POPC-cholesterol bilayers respectively.

To discard the difficulty of tau (time constant) evaluation online, the planar lipid bilayer capacitance was calculated from the modified equation: C*_m_* = t/R*_p_*ln(I_0_/I_t_) [18] where t = time in seconds, I_0_ is the steady state current at t = 0, I_t_ is the current at time t, and R*_p_* is the leakage resistance given by R*_p_* = R_m_R_i_/R_m_+R_i_ for potentials from 30–60 mV. R*_p_* was included in the Cm equation and extrapolated for the complete potential range (30–180 mV). Cm decreased with increased potentials for all bilayers in a bell-shape behavior.

#### Analysis programme

The resistance is calculated according to Ohm's law
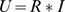
(1)i.e.
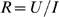
(2)Which is normalized to the membrane area
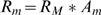
(3)


To calculate the membrane capacitance (see below) the term R*_p_* (leakage resistance) was obtained from:
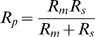
(4)where R*_m_* comes from equation (3) and R*_s_* is the series resistance given by the resistance of the glass micropipette (1 MΩ)

### Statistical data and consequences on mean and standard error for lipid bilayer capacitance

#### Current traces averaging and standard error

Average traces are calculated for six different files obtained for a same voltage step. To each point is associated the mean and standard error. The mean is calculated from:
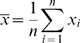



And standard error from:
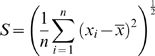



### Stat reminder

Two data series a and b with a mean of
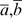



And standard error
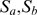



1. Mean and standard error of a sum

Then the data series y = a+b has a mean of
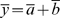



And standard error
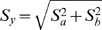



2. Mean and standard error of a product and a quotient

The data series y = a * b ou y = a/b has a mean of
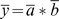
Or
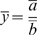



And standard error of
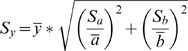



3. Logarithm


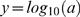
 has a mean of 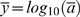 and standard error
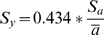



### Mean and standard error for lipid bilayer capacitance



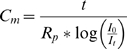
(5)Modified from Ti Tien [18]

Given 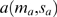, 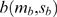, 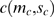, 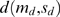


Then it comes: 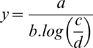


Which has a standard error of:
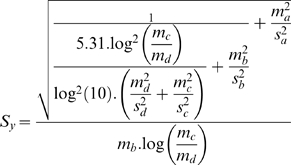



Calculated with maxima-5.5 (created by W. Schelter;http://ww3.ac-poitiers.fr/math/prof/logic/gos6/)

And a mean of : 
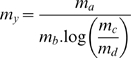


Formulas were checked using Matlab and test vectors of known mean and standard errors.

Transport of anandamide through a reconstituted planar lipid bilayer

Kinetics of 1 µmole anandamide transport across planar lipid bilayers of controled lipid composition were carried out at 20°C using planar lipid bilayer chambers from Warner Instrument as described above. The anandamide transport is measured with an anandamide assay at various times using 5-lipoxygenase from soybean. An excess of 5-lipoxygenase (50.000 units, type I-B Sigma) catalyses at 20°C in 1.1 ml of 0.1 M carbonate/bicarbonate buffer, 2% Tween 20, (Sigma), pH 9, the complete hydroperoxydation of lipids containing cis-cis pentadiene structure as linoleic acid, arachidonic acid or anandamide [23]. The reaction is followed by measuring the increase in absorbance at 234 nm against lipoxygenase solution in 2% Tween 20. One enzymatic unit causes an increase in absorbance at 234 nm of 0.001 per minute at 20°C at pH 9 when linoleic acid is used as substrate. The soybean 5-lipoxygenase uses anandamide as substrate with approximately 15% of the activity indicated using linoleic acid. The amount of anandamide accumulated in the trans compartment was estimated considering that one absorbance unit at 234 nm is equivalent to the oxidation 0.12 micromole of anandamide, on the basis of a calibration curve.

Experimental data were analysed with the Origin programme, version 3.5 (Microcal software). The Boltzman (x,A1,A2,x0,dx) function producing a sigmoidal curve was used according to the equation:

(A1–A2)/[1+exp((x−x0)/dx)]+A2 with parameters of x0 (center, i.e., x at y50), dx (width), A1 (Y initial), and A2 (Y final).
